# Technology-Enabled, Evidence-Driven, and Patient-Centered: The Way Forward for Regulating Software as a Medical Device

**DOI:** 10.2196/34038

**Published:** 2022-01-27

**Authors:** Jane Elizabeth Carolan, John McGonigle, Andrea Dennis, Paula Lorgelly, Amitava Banerjee

**Affiliations:** 1 Institute of Health Informatics University College London London United Kingdom; 2 Institute of Epidemiology and Health Care University College London London United Kingdom; 3 Perspectum Ltd Oxford United Kingdom; 4 University College London Hospitals NHS Foundation Trust London United Kingdom; 5 Barts Health NHS Trust London United Kingdom

**Keywords:** Artificial intelligence, machine learning, algorithm, software, risk assessment, informatics

## Abstract

Artificial intelligence (AI) is a broad discipline that aims to understand and design systems that display properties of intelligence. Machine learning (ML) is a subset of AI that describes how algorithms and models can assist computer systems in progressively improving their performance. In health care, an increasingly common application of AI/ML is software as a medical device (SaMD), which has the intention to diagnose, treat, cure, mitigate, or prevent disease. AI/ML includes either “locked” or “continuous learning” algorithms. Locked algorithms consistently provide the same output for a particular input. Conversely, continuous learning algorithms, in their infancy in terms of SaMD, modify in real-time based on incoming real-world data, without controlled software version releases. This continuous learning has the potential to better handle local population characteristics, but with the risk of reinforcing existing structural biases. Continuous learning algorithms pose the greatest regulatory complexity, requiring seemingly continuous oversight in the form of special controls to ensure ongoing safety and effectiveness. We describe the challenges of continuous learning algorithms, then highlight the new evidence standards and frameworks under development, and discuss the need for stakeholder engagement. The paper concludes with 2 key steps that regulators need to address in order to optimize and realize the benefits of SaMD: first, international standards and guiding principles addressing the uniqueness of SaMD with a continuous learning algorithm are required and second, throughout the product life cycle and appropriate to the SaMD risk classification, there needs to be continuous communication between regulators, developers, and SaMD end users to ensure vigilance and an accurate understanding of the technology.

## Introduction

Artificial intelligence (AI) is a broad discipline that aims to understand and design systems that display properties of intelligence [1]. Machine learning (ML) is a subset of AI that describes how algorithms and models can assist computer systems in progressively improving their performance [2]. Based on publicly available information, in late September 2021, the US Food and Drug Administration (FDA) listed (noting “initial list” only) 343 AI/ML-enabled medical devices marketed in the United States. In health care, an increasingly common application of AI and ML is software as a medical device (SaMD), which has the intention to diagnose, treat, cure, mitigate, or prevent disease [3]. Regulatory frameworks for SaMD need to be adaptive while prioritizing patient safety and effectiveness [4-6]. Regulatory challenges of SaMD include processing submitted evidence to verify clinical effectiveness, generalizability, interoperability, data integrity, and data security. Constructing a fit-for-purpose regulatory framework for SaMD with a continuous learning algorithm is an added complexity. As regulatory agencies aim to advance health care delivery through SaMD adoption, with efforts to avoid unintended consequences, this commentary summarizes the current regulatory frameworks for SaMD. First, we describe the challenges of continuous learning algorithms, then highlight the new evidence standards and frameworks under development, and discuss the need for stakeholder engagement, concluding with 2 key steps that regulators need to address in order to optimize and realize the many benefits of SaMD.

## Technology-Enabled Algorithms

ML techniques incorporate training, validation, and test data sets at different stages of model development. Algorithms are executed in a training data set and results compared with a target value. Parameters of the model are adjusted accordingly as part of this process. Identifying potential data biases (including age, ethnicity, vendor, disease prevalence) is critical, but not limited to this point. At the validation stage, the fitted model is used to predict responses for observations in the validation data set, a process of fine-tuning the model. In the test stage, the ML model is exposed to a test data set, independent of training or validation data sets, providing unbiased evaluation of the final model. AI/ML includes either “locked” or “continuous learning” algorithms. Locked algorithms consistently provide the same output for a particular input. Such algorithms may be modified to optimize performance, requiring “episodic” regulatory review if the algorithm requires additional inputs or changes in intended use or performance. Continuous learning algorithms, in their infancy in terms of SaMD, modify in real-time based on incoming real-world data, without controlled software version releases. Continuous learning algorithms pose the greatest regulatory complexity, requiring seemingly continuous oversight in the form of special controls to ensure ongoing safety and effectiveness.

Although systems with continuous learning may appear conceptually similar to systems that self-calibrate to the local environment (eg, adapting to temperature), continuous learning algorithms using modern ML techniques are qualitatively different in that portions of their algorithms, in the form of their trained networks, are being modified autonomously. This continuous learning has the potential to better handle local population characteristics, but with the risk of reinforcing existing structural biases, potentially without adequate oversight. Thus, special regulations are needed to classify these risks and accordingly, ensure appropriate human oversight.

## Frameworks and Standards for the Future

Medical device regulatory agencies such as the US FDA, EU Notified Bodies, and the UK Medicines and Healthcare products Regulatory Agency (MHRA) have responsibility for protecting public health by only enabling market access for safe and effective products. Further down the line, importantly, health care budget holders then need to assess cost-effectiveness and budget impact, a potential rate-limiting step for successful market access. Lessons on successful AI/ML adoption in other industries are limited in their value given the unique health risks and benefits that health care regulators must assess. To verify claims of safety and effectiveness in the form of submitted evidence, regulators must keep pace with the complexity of algorithm models, including validation and testing stages, selected use of software of unknown pedigree, and real-world performance [7].

The FDA has outlined its proposed framework for SaMD in a total product life cycle approach [4] and released an AI/ML-based SaMD action plan [8] in response to stakeholder feedback. At the premarket submission stage, a predetermined change control plan would play a role in obtaining reasonable assurance of safety and effectiveness: developers would stipulate what anticipated algorithm modifications would occur, and how the algorithm would learn and change without compromising safety or performance. Postmarket access, periodic updates to the FDA on changes to the algorithm to enable ongoing oversight of real-world performance would be provided. Early next year, draft guidance on detailed requirements is anticipated; currently, it is not evident how much oversight should be performed by the end user(s) and manufacturer, nor how much robust data are needed to substantiate safety and effectiveness claims.

To promote rigor and transparency in design and reporting of AI-based interventions (underpinning regulatory submission evidence claims), reporting guidelines and checklists include Consolidated Standards of Reporting Trials–Artificial Intelligence (CONSORT-AI), Standard Protocol Items: Recommendations for Interventional Trials–Artificial Intelligence (SPIRIT-AI), The Transparent Reporting of a multivariable prediction model of Individual Prognosis Or Diagnosis-Artificial Intelligence (TRIPOD-AI), and Minimum Information About Clinical Artificial Intelligence Modeling (MI-CLAIM) [9,10]. In the UK, the National Institute for Health and Care Excellence (NICE) has also released revised evidence standards for digital health technologies [11]. Currently, there is an absence of tailored frameworks for AI/ML-based SaMD with a continuous learning algorithm; guidelines including MI-CLAIM and NICE’s evidence standards framework, while valuable for locked algorithms, note that continuous learning algorithms are beyond their scope.

Globally, the International Medical Device Regulators Federation (IMDRF) aims to accelerate medical device international regulatory harmonization and has drafted key SaMD policies to complement existing international standards, particularly in terms of risk classification, converging terminology, a risk-based framework, and quality management systems. The Institute of Electrical and Electronic Engineers (IEEE) has Artificial Intelligence Medical Device Working Groups on terminology and recommended practice for the quality management of data sets. United Nations agency collaboration between the World Health Organization and the International Telecommunication Union: Focus Group on Artificial Intelligence for Health (FG-AI4H) was established to use AI to advance health care for all, and to benchmark AI models using secure and confidential, globally representative data sets [12].

## The Need for Stakeholder Engagement

It is recognized that patient-centered data and engagement play a fundamental role in regulatory assessment of SaMD. The “patient-centered” approach referred to by the FDA addresses usability, equity, trust, and accountability. Engagement with both developers and end users occurred at a February 2020 Public Workshop on the Evolving Role of Artificial Intelligence in Radiological Imaging. At the latter event, The American College of Radiology (ACR) and Radiological Society of North America (RSNA) questioned [13] the ability of the FDA to ensure safety and effectiveness of continuous learning algorithms, without direct physician or expert oversight during each use. Familiar concerns relate to autonomous image interpretation independent of physician confirmation and oversight. If an algorithm ceases to function properly without radiologist oversight, a significant number of patients are at risk of incorrect screening before algorithm failure is recognized. It was noted that algorithm user manuals must have clear guidance regarding which equipment and protocols are supported, and deployment restricted to those settings studied during validation. Evaluation of real-world algorithm performance will reassure patients and health professionals of readiness for clinical use.

## Conclusion

SaMD has great potential to improve health and health care at individual and system levels. To optimize on the benefits associated with SaMD, patient safety and effectiveness need to be aptly assessed for which 2 key steps are necessary. First, international standards and guiding principles addressing the uniqueness of SaMD with a continuous learning algorithm are required [14], outlining best practice oversight and reporting requirements. Aligned regulatory requirements, tailor-made for SaMD with a continuous learning algorithm, are essential, particularly to verify maintenance measures to keep in check modifications throughout the life cycle of SaMD. A special registry dedicated to these technologies may also be appropriate. Depending on the degree of risk to patients from a particular application of AI/ML SaMD, a degree of expert clinical oversight coupled with technology industry/developer assurance is likely to be required. Second, throughout the product life cycle, appropriate to the risk classification of the SaMD product, there needs to be continuous communication between regulators, developers, and SaMD end users to ensure vigilance and an accurate understanding of the technology. The latter will facilitate the adoption of state-of-the-art automation, optimizing clinical effectiveness and ensuring patient safety.

## Abbreviations

ACR: American College of Radiology
AI: artificial intelligence
CONSORT-AI: Consolidated Standards of Reporting Trials–Artificial Intelligence
FDA: the US Food and Drug Administration
FG-AI4H: Focus Group on Artificial Intelligence for Health
IEEE: Institute of Electrical and Electronic Engineers
IMDRF: International Medical Device Regulators Federation
MHRA: the UK Medicines and Healthcare products Regulatory Agency
MI-CLAIM: Minimum Information About Clinical Artificial Intelligence Modeling
ML: machine learning
NICE: National Institute for Health and Care Excellence
RSNA: Radiological Society of North America
SaMD: software as a medical device
SPIRIT-AI: Standard Protocol Items: Recommendations for Interventional Trials–Artificial Intelligence
TRIPOD-AI: The Transparent Reporting of a multivariable prediction model of Individual Prognosis Or Diagnosis- Artificial Intelligence
